# Factors influencing mortality in patients with abdominal trauma undergoing damage control therapy

**DOI:** 10.1590/0100-6991e-2026004425-en

**Published:** 2026-05-22

**Authors:** FLÁVIA CASTANHO HUBERT, FERNANDA BAEUMLE REESE, MARIANA BRUINJE COSENTINO, MARIA ISABEL OZUNA DOS SANTOS, RAFAELLA STRADIOTTO BERNARDELLI, JORGE EDUARDO FOUTO MATIAS

**Affiliations:** 1 - Hospital de Clínicas da Universidade Federal do Paraná (Unidade de Terapia Intensiva Adulto - UTIAD) - Curitiba - PR - Brasil; 2 - Unidersidade Federal do Paraná - Curitiba - PR - Brasil; 3 - Pontifica Universidade Católica do Paraná - Curitiba - PR - Brasil

**Keywords:** Trauma surgery, Abdominal injuries, Trauma Severity Indices, Laparotomy, Mortality, Cirurgia de Cuidados Críticos, Traumatismos Abdominais Índices de Gravidade do Trauma, Laparotomia, Mortalidade

## Abstract

**Objectives:**

Damage control surgery aims to reduce mortality in patients with severe abdominal trauma by limiting prolonged interventions under unfavorable physiological conditions. This study aimed to identify factors associated with mortality in these patients, including sex, age, cause and mechanism of injury, trauma severity index, hypothermia, metabolic acidosis, and variables related to intensive care, anesthesia, surgical management, and laboratory findings at admission to the intensive care unit (ICU).

**Methods:**

This retrospective observational cohort study analyzed 136 medical records of patients with abdominal trauma who underwent damage control surgery at a tertiary trauma hospital in Curitiba, Paraná, Brazil. Clinical, surgical, and laboratory variables were evaluated. Survivors and non-survivors were compared using univariate and multivariate statistical analyses to determine the significance of the findings.

**Results:**

Relevant findings included higher trauma severity scores among patients who died (mean Injury Severity Score [ISS] of 34 in non-survivors vs 29 in survivors; p=0.017). Hypothermia at admission was significantly associated with mortality. Metabolic acidosis at ICU admission was also associated with unfavorable outcomes, represented by a low bicarbonato value as well as a low base excess (BE) value of -12 compared with -8.6 (p<0.001).

**Conclusions:**

Higher ISS values, more negative BE, and hypothermia were independent predictors of mortality in patients undergoing damage control therapy for abdominal trauma.

## RESUMO

**Objetivos:**

A cirurgia de controle de danos tem como finalidade reduzir a mortalidade em vítimas de trauma abdominal grave por meio da limitação de intervenções prolongadas em condições fisiológicas desfavoráveis. Este estudo teve como objetivo identificar os fatores associados à mortalidade nesses pacientes, considerando variáveis como sexo, idade, causa e mecanismo do trauma, índice de gravidade do trauma, hipotermia e acidose metabólica, além de fatores relacionados aos cuidados intensivos, anestésicos, cirúrgicos e resultados laboratoriais da admissão na unidade de terapia intensiva.

**Métodos:**

Estudo observacional retrospectivo de coorte histórica, realizado por meio da análise de 136 prontuários de pacientes com trauma abdominal submetidos à cirurgia de controle de danos em hospital terciário de trauma, em Curitiba-PR. Foram analisadas variáveis clínicas, cirúrgicas e laboratoriais, comparando-se os grupos de sobreviventes e não sobreviventes utilizando testes estatísticos univariados e multivariados para determinar a significância dos resultados.

**Resultados:**

Dentre os dados relevantes destacam-se a pontuação no escore de trauma entre pacientes que evoluíram a óbito (ISS médio de 34 nos falecidos e 29 nos sobreviventes, p=0,017). Além disso, hipotermia na admissão mostrou correlação significativa com a mortalidade, bem como acidose metabólica na admissão da UTI (representada por bicarbonato baixo, bem como por valor de excesso de bases (BE) de -12 contra -8,6 <0,001) com desfecho desfavorável.

**Conclusão:**

O valor do ISS mais elevado, bem como BE mais negativo e hipotermia são preditores independentes de mortalidade em pacientes submetidos a terapia de controle de danos por trauma abdominal.

## INTRODUCTION

Trauma is one of the leading causes of death during the first half of life. Road traffic accidents and violence are classified as external causes, and it is estimated that more than 5.8 million people die each year due to accidents and violence, according to World Health Organization data^1^.

Among fatal injuries, abdominal trauma is present in approximately 13%-15% of cases. Its relevance is related both to its high incidence and to the frequent need for immediate diagnosis and surgical intervention. In the most severe cases, damage control therapy has become an essential strategy because it shortens the initial operative time, interrupts the progression of potentially fatal pathophysiological processes, and allows patient stabilization before definitive management^2^.

Before the implementation of this technique, mortality rates among patients with severe abdominal trauma were higher, mainly due to prolonged operative time, which favored the development of coagulopathy, metabolic acidosis, tissue hypoperfusion, hemodynamic instability, infections, and respiratory complications^3^.

The main methods of abbreviated laparotomy, or damage control surgery, to achieve hemorrhage control include ligation, suturing, or temporary vascular shunting, packing of hepatic injuries, and splenectomy in the presence of splenic injury^3^. Subsequently, during the stabilization phase, control of bleeding, hypothermia, acidosis, tissue hypoperfusion, and metabolic disturbances becomes a priority, aiming to prevent progression to the so-called lethal triad.

The management of polytrauma patients is challenged by the wide variability in the nature and severity of injuries. This heterogeneity also affects study design and the standardization of analyzed groups. A relevant strategy is the use of trauma scoring systems, which allow patients to be stratified according to the severity of their injuries^4^.

Understanding the relationships among factors such as length of hospital stay, mechanism of injury, severity, coagulation disorders, and hypoperfusion is essential to guide more effective interventions in patients undergoing damage control therapy. This analysis provides evidence directly applicable to intensive care management, supporting a more precise approach aligned with individual patient needs.

This study aimed to identify factors associated with mortality in patients with abdominal trauma undergoing damage control therapy by evaluating general variables (sex, age, cause and mechanism of injury), severity indices, parameters related to hypothermia and metabolic acidosis as well as anesthetic, surgical, intensive care unit (ICU) variables and laboratory findings at admission to the ICU. This initial analysis may support future prospective studies to further investigate these associations and strengthen the understanding of the lethal triad within a Brazilian population of patients with severe trauma.

## METHODS

The study was approved by the human research ethics committee (No. 2,751,732) and did not involve additional interventions.

This historical cohort study was based on the analysis of medical records of patients with abdominal trauma who underwent damage control therapy between January 2012 and December 2018 at a trauma referral hospital in the state of Paraná, Brazil. As a retrospective study, a convenience sample was used, including all available cases during the study period. Missing data were not imputed, and analyses were conducted only for cases with complete information.

Adult patients (≥18 years) who underwent damage control therapy with peritoneostomy for traumatic indications were included. Exclusion criteria were age <18 years, pregnancy, non-abdominal trauma, absence of initial arterial blood gas analysis, and cases in which laparotomy could not be classified as damage control. Hypothermia was defined as axillary temperature <36 °C. Temperatures were measured using an axillary thermometer, and data were obtained from nursing admission records.

The following variables were collected and entered into electronic spreadsheets: admission date, sex, age, cause of injury (gunshot wound, stab wound, traffic accident, or fall), trauma mechanism (penetrating or blunt), emergency department admission time, associated injuries, comorbidities, trauma scores (Abbreviated Injury Scale, Injury Severity Score [ISS], Revised Trauma Score, Trauma Score and Injury Severity Score [TRISS]), arterial blood gas parameters at admission (pH, bicarbonate, base excess, and lactate), and coagulation test results at admission (prothrombin time and activated partial thromboplastin time).

Additional variables included tranexamic acid administration, volume of colloid or crystalloid infusion, use of negative pressure therapy in peritoneostomy, time to first reoperation, type of abdominal closure, time to abdominal closure, number of days of vasoactive drug use, duration of mechanical ventilation, duration of sedation, and length of hospital stay. The occurrence of complications, need for hemodialysis during hospitalization, and final outcome (discharge or death) were also recorded.

Results for each parameter were expressed as means for the subgroups of patients who died and those discharged from the hospital. Comparative statistical analysis was performed to determine whether significant differences existed between groups. Both univariate and multivariate analyses were conducted.

Categorical variables were described using absolute and relative frequencies, whereas numerical variables were described using mean, standard deviation, median, minimum, and maximum values. Associations between ICU mortality (discharge vs death) and dichotomous categorical variables were assessed using Fisher’s exact test, while trichotomous categorical variables were analyzed using the chi-square test. Differences in numerical variables between discharge and death outcomes were analyzed using the Student t test when normal distribution was confirmed by the Kolmogorov-Smirnov test. For variables without normal distribution, the nonparametric Mann-Whitney test was applied.

Multivariate analysis was presented using two complementary models to highlight multicollinearity (variables with similar clinical and statistical meaning), which may lead to exclusion of closely related variables. Multicollinearity was observed between bicarbonate and base excess (BE) values as well as between cause of trauma (gunshot vs stab wound) and trauma mechanism, for example.

Statistical analysis was performed using Stata software, and statistical significance was set at 5% (p<0.05).

Because this was a retrospective study, the statistical analyses were appropriate for the proposed design. However, the relatively small sample size represents an important limitation and should be considered when interpreting the results. The limited number of cases reduces statistical power and the ability to detect subtle differences between groups, and therefore should be explicitly recognized as a methodological limitation.

## RESULTS

A total of 136 patients were evaluated, including 121 men (89%) and 15 women (11%). Overall mortality in the study population was 33%.

Overall characteristics of the sample, including clinical and laboratory profiles, are presented in Table 1. A descriptive overview of variables related to management and clinical course is presented in Table 2.

**Table 1:** General description of the sample (clinical and laboratory profile).

Variable	Descriptive
Sex	
Men	121 (89%)
Women	15 (11%)
Age, years	32.5 ± 11.9; 30 (18-68)
Mechanism of injury (n = 136)	
Gunshot wound	74 (54.4%)
Stab wound	18 (13.2%)
Traffic accident	35 (25.7%)
Falls	9 (6.6%)
Variable	Descriptive
Type of trauma (n = 136)	
Penetrating	93 (68.4%)
Blunt	43 (31.6%)
ISS (n = 136)	30.5 ± 10.8; 31 (9-66)
TRISS (n = 136)	84.5 ± 22.5; 94.2 (6.5-99.1)
Hypothermia (n = 136)	79 (59%)
pH (n = 136)	7.23 ± 0.1; 7.24 (6.86-7.45)
Bicarbonate (n = 136)	16.7 ± 3.4; 17.1 (6-24.9)
Base excess (n = 136)	−10.15 ± 4.5; −9.3 (−26.5 to −0.3)
Lactate (n = 136)	4.8 ± 2.9; 4 (0.6-15.3)
Prothrombin activity time (n = 117)	1.3 ± 0.31; 1.23 (0.95-2.8)
Activated partial thromboplastin time (n = 117)	36.1 ± 26.2; 30.2 (15.9-180)

ISS: Injury Severity Score; TRISS: Trauma and Injury Severity Score.

Analysis of Tables 1 and 2 shows a predominance of adult male patients with a mean age of 32 years. The sample was characterized by a mean ISS of 30.5 as well as metabolic acidosis at admission and clinical signs consistent with hypoperfusion.

**Table 2:** General description of the sample (management and clinical course)

Variable	Descriptive
Tranexamic acid (n = 136)	61 (44.9%)
Colloid volume in the operating room (n = 136)	29.4 ± 169.6; 0 (0-1500)
Crystalloid volume in the operating room (n = 134)	2741 ± 2540; 2500 (0-13,500)
Colloid volume during the first 24 h in the ICU (n = 131)	215.1 ± 748.5; 0 (0-6000)
Crystalloid volume during the first 24 h in the ICU (n = 133)	4298.9 ± 2248.8; 4000 (0-10,500)
Fluid balance during the first 24 h in the ICU (n = 134)	9103.15 ± 5289.3; 8375 (1742-48,886)
Negative pressure therapy in peritoneostomy (n = 136)	
No vacuum	64 (47.1%)
Vacuum at first procedure	29 (21.3%)
Variable	Descriptive
Vacuum at any procedure	43 (31.6%)
Time to first reoperation (n = 123)	
1 to 3 days	119 (96.7%)
4 days	4 (3.3%)
Days using vasoactive drugs (n = 135)	4.9 ± 3.5; 4 (0-23)
Days of mechanical ventilation (n = 135)	11.7 ± 10.6; 7 (0-49)
Days of sedation (n = 136)	5.2 ± 5.4; 4 (0-33)
Length of hospital stay (n = 136)	21.6 ± 23.1; 13.5 (0-141)
Hemodialysis (n = 136)	30 (22.1%)
Infection (n = 136)	92 (67.6%)
Peritoneostomy complications (n = 136)	69 (50.7%)
Mortality (n = 136)	45 (33%)

Categorical variables are presented as absolute frequency (percentage), whereas numerical variables are described as mean ± standard deviation and median (minimum-maximum). n represents the sample size considered for each variable. ICU: intensive care unit.

Table 3 presents comparisons of variables between patients discharged from the ICU and those who died.

**Table 3:** Comparison of general variables between patients discharged from the ICU (n = 91) and those who died (n = 45).

Variável	ICU discharge (n = 91)	ICU death (n = 45)	p-value
Sex			0.773***
Men	80 (87.9%)	41 (91.1%)	
Women	11 (12.1%)	4 (8.9%)	
Age, years	30.2 ± 10; 28 (18-62)	37.3 ± 14; 35 (18-68)	0.370*
Mechanism of injury			0.105****
Gunshot wound	52 (57.1%)	22 (48.9%)	
Stab wound	15 (16.5%)	3 (6.7%)	
Traffic accident	20 (22%)	15 (33.3%)	
Falls	4 (4.4%)	5 (11.1%)	
Type of trauma			0.780***
Penetrating	67 (73.6%)	26 (57.8%)	
Blunt	24 (26.4%)	19 (42.2%)	
ISS	29 ± 10.8; 29 (9-66)	33.7 ± 10.1; 34 (13-54)	0.017*
TRISS	87 ± 21.1; 95 (6.5-99.1)	79.3 ± 24.5; 89.4 (11.1-98.8)	0.004**

Categorical variables are presented as absolute frequency (percentage), whereas numerical variables are described as mean ± standard deviation and median (minimum-maximum). n represents the sample size considered for each variable.*Student t test; **Mann-Whitney test; ***Fisher exact test; ****Kruskal-Wallis test. Statistical significance was set at 5% (p < 0.05). ICU: intensive care unit; ISS: Injury Severity Score; TRISS: Trauma and Injury Severity Score.

No significant differences were observed between survivors and non-survivors regarding age, sex, cause, or mechanism of injury. However, among patients who died, a higher mean ISS of 33.7 and a TRISS of 79.3 were notable.

The main findings from the univariate and multivariate analyses are presented in Table 4.

**Table 4:** Univariate and multivariate analysis of variables related to acidosis and coagulopathy and their comparison between patients discharged from the ICU (n = 91) and those who died (n = 45).

Model	Variable	n	OR (95% CI)	p-value
Univariate logistic regression for mortality				
	pH at ICU admission	136	0.002 (0.000-0.096)	0.001
	Bicarbonate at ICU admission	136	0.75 (0.65-0.85)	< 0.001
	BE at ICU admission	136	0.815 (0.74-0.90)	< 0.001
	Lactate at ICU admission	136	1.33 (1.15-1.54)	< 0.001
	Prothrombin activity time at ICU admission	117	4.30 (1.15-16.11)	0.031
	Activated partial thromboplastin time at ICU admission	117	1.00 (1.00-1.04)	0.051
Multivariate logistic regression - most adjusted model (forward stepwise - conditional)				
	Bicarbonate at ICU admission	115	0.76 (0.66-0.875)	< 0.001
Multivariate logistic regression - most adjusted model (backward stepwise - conditional)				
	BE at ICU admission	115	0.82 (0.74-0.91)	< 0.001
Multivariate logistic regression - most adjusted model (backward stepwise - conditional)				
	Hypothermia during hospitalization (no hypothermia as reference)	-	3.11 (1.16-8.41)	0.025

BE: base excess; ICU: intensive care unit; OR: odds ratio.

In the univariate analysis, the variables associated with mortality included acidosis (pH and bicarbonate), more negative BE, elevated lactate levels, and coagulation abnormalities.

However, in the multivariate analysis, only lower bicarbonate values, more negative BE, and hypothermia remained significant, suggesting an association with increased mortality.

The influence of hypothermia and the need for hemodialysis on mortality, assessed through both univariate and multivariate analyses, is presented in Table 5.

**Table 5:** Comparison of hypothermia and hemodialysis variables between patients discharged from the ICU (n = 91) and those who died (n = 45): univariate and multivariate analyses.

Model	Variable	n	OR (95% CI)	p-value
Univariate logistic regression for mortality				
	Hypothermia (absence as reference)	134	1.63 (0.77-3.45)	0.199
	Hemodialysis (no hemodialysis as reference)	136	7.97 (3.23-19.68)	< 0.001
Model	Variable	n	OR (95% CI)	p-value
Multivariate logistic regression (enter method; variables with p < 0.25)				
	Hypothermia during hospitalization	127	3.17 (1.02-9.83)	0.046
	Hemodialysis during hospitalization	127	39.49 (8.49-183.67)	< 0.001
Multivariate logistic regression (enter method; variables with p < 0.25)				
	Hypothermia during hospitalization (absence as reference)	134	3.11 (1.16-8.41)	0.025
	Hemodialysis during hospitalization (no hemodialysis as reference)	134	52.07 (10.59-255.94)	< 0.001

ICU: intensive care unit; OR: odds ratio.

A consistent trend toward increased mortality was observed among patients who developed hypothermia during hospitalization, with an OR of 3.11 (p < 0.001). The need for hemodialysis also showed a strong association with death, with a mortality rate of 46.07% and an OR of 52.07.

## DISCUSSION

The mortality observed in this study, 33% among patients admitted to the ICU after damage control surgery, is consistent with values reported in prior research, which range from 26% to 67%^5^. Mortality was slightly lower among women (26.6%) and higher among men (33.8%), a pattern that reflects the well-established distribution of external causes. According to Silveira & O’Dwyer1, approximately 83% of deaths from external causes occur in men, reinforcing the male predominance observed in our sample.

The main cause of abdominal trauma requiring damage control was gunshot injury (54.4%), a finding consistent with other published series^1^.

The analysis presented in this study is based on a population with extremely high injury severity. The mean ISS was 30.5, substantially above the threshold for severe polytrauma (ISS >15)^6^. In comparison, a 2016 study evaluating 1,030 trauma laparotomies reported a mean ISS of 16.7 Another analysis including 1,561 patients confirmed the association between higher trauma scores as well as age above 45 years, and increased mortality^8^.

In our study, patients discharged from the ICU had a mean ISS of 29, whereas those who died had a mean ISS of 34. Multivariate analysis demonstrated that this difference indicates the score as an independent predictor of mortality. International studies similarly support the role of ISS as a predictor of outcomes in abdominal trauma. For example, a study conducted in Nigeria reported a mean ISS of 15 among patients with favorable outcomes and 23 among those with unfavorable outcomes^9^.

In multivariate analyses, the TRISS score did not show statistically significant differences, while the need for hemodialysis emerged as an important marker of severity.

The mean BE of −10.15 at ICU admission was identified as a predictor of mortality, with an OR of 0.82 (95% CI, 0.74-0.91; p <0.001). Both BE and arterial lactate reflect acidosis associated with tissue hypoperfusion, and research describes their relationship with increased mortality within the first 24h^10^. Increased lactate and more negative BE values are associated with a higher risk of multiple organ dysfunction in polytrauma patients. A European study conducted in 2014 involving 255 trauma patients found significant differences in BE values between patients with more severe shock compared with those with milder shock (classes I and II). Mean BE values were −3.4 in classes I and II and −6.9 in classes III and IV, and the study also reported higher mortality and ISS values among patients with class III and IV shock^11^.

Another independent predictor of mortality was the presence of hypothermia during hospitalization, with an OR of 3.11 and p <0.001 in multivariate analysis. The literature consistently identifies hypothermia at admission as a risk factor for mortality after trauma. A US study evaluating 1,252 trauma patients requiring cavity surgery reported that 15% were admitted with a temperature below 35°C. Compared with normothermic patients at the end of surgery, hypothermic patients had significantly higher mortality (35% vs 8%)^12^. Risk factors for hypothermia in trauma include injury severity, anesthesia (including prehospital), intubation, low ambient temperature, wet clothing, administration of cold fluids, environmental exposure, and hypovolemic shock, often resulting from a combination of these factors^13^.

This study has several limitations, primarily related to its retrospective design, incomplete data, lack of standardized resuscitation protocols, and limited control over management variables. Nevertheless, these findings may contribute to future prospective studies aimed at confirming the associations observed here.

## CONCLUSION

The analysis of 136 patients who underwent damage control surgery with peritoneostomy in this study demonstrated that the sample consisted of individuals with extremely high injury severity, reflected by a mean ISS of 30. Higher ISS, more negative BE, and hypothermia were identified as independent predictors of mortality, reinforcing the role of these markers in the early identification of metabolic decompensation and unfavorable prognosis. Although arterial blood gas analysis appeared to be a potential prognostic indicator, its clinical use requires prospective validation.

Hemodialysis showed a strong association with mortality; however, this association may primarily reflect greater clinical severity among patients rather than a direct causal effect.

This study has limitations inherent to its retrospective design, including a relatively small sample size, limited statistical power, and structural constraints related to the collection of clinical and laboratory data. Nevertheless, the findings contribute to improving the understanding of prognostic factors following damage control therapy, particularly in the intensive care setting, supporting the identification of high-risk patients and guiding more individualized care strategies.

## References

[1] Silveira E da S.O'Dwyer G (2017). Centro de Trauma modelo alternativo de atendimento às causas externas no estado do Rio de Janeiro. Saúde debate.

[2] Neves A de S.Carneiro PR.Miranda D de CS.Vieira HE.Abrantes WL (2016). Damage control surgery in the abdominal trauma. Rev Médica Minas Gerais.

[3] Cirocchi R, Montedori A, Farinella E, Bonacini I, Tagliabue L, Abraha I (2013). Damage control surgery for abdominal trauma. Cochrane Database Syst Rev.

[4] Yong Jin WY, Jeong JH, Kim DH, Kim TY, Kang C, Lee SH (2018). Factors predicting the early mortality of trauma patients. Ulus Travma ve Acil Cerrahi Derg.

[5] Timmermans J, Andrew N, Kairinos N, Teijink J, Prins M, Navsaria P (2010). Predicting mortality in damage control surgery for major abdominal trauma. South African J Surg.

[6] Sousa AN, Paiva JA, Fonseca SA, Raposo FJ, Loureiro ÁM, Valente LF (2011). Trauma Scores na Avaliação de Politraumatizados Quais e Para Quê?. Acta Med Port.

[7] Joseph B, Azim A, Zangbar B, Bauman Z, O'Keeffe T.Ibraheem K (2016). Improving mortality in trauma laparotomy through the evolution of damage control resuscitation Analysis of 1,030 consecutive trauma laparotomies. J Trauma Acute Care Surg.

[8] Chiang YT, Lin TH, Hu RH, Lee PC, Shih HC (2021). Predicting factors for major trauma patient mortality analyzed from trauma registry system. Asian J Surg.

[9] Williams-Johnson JA, McDonald AH, Strachan GG, Williams EW (2010). Effects of tranexamic acid on death, vascular occlusive events, and blood transfusion in trauma patients with significant haemorrhage (CRASH-2) A randomised, placebo-controlled trial. West Indian Med J.

[10] Sabogal CEL, Rivera AFC, Higuera AYJ (2014). Lactate and base deficit in trauma Prognostic value. Rev Colomb Anestesiol.

[11] Evers MJ, Vaneker M, Biert J (2014). Polytrauma at the Emergency Department; can we relate arterial blood gas analysis to a shock classification. Eur J Trauma Emerg Surg.

[12] Inaba K, Teixeira PGR, Rhee P, Brown C, Salim A, Dubose J (2009). Mortality impact of hypothermia after cavitary explorations in trauma. World J Surg.

[13] Van Veelen MJ, Brodmann Maeder M (2021). Hypothermia in trauma. Int J Environ Res Public Health.

